# Efficacy of the antimicrobial peptide TP4 against *Helicobacter pylori* infection: *in vitro* membrane perturbation *via* micellization and *in vivo* suppression of host immune responses in a mouse model

**DOI:** 10.18632/oncotarget.4101

**Published:** 2015-05-11

**Authors:** Jayaram Lakshmaiah Narayana, Han-Ning Huang, Chang-Jer Wu, Jyh-Yih Chen

**Affiliations:** ^1^ Doctoral Degree Program in Marine Biotechnology, Academia Sinica and National Sun Yat-sen University, Kaohsiung, Taiwan; ^2^ Marine Research Station, Institute of Cellular and Orgasmic Biology, Academia Sinica, Jiaushi, Ilan, Taiwan; ^3^ Department of Food Science, National Taiwan Ocean University, Keelung, Taiwan

**Keywords:** Helicobacter pylori, antimicrobial peptide, tilapia piscidin 4 (TP4), micellization

## Abstract

*Helicobacter pylori* infection is marked by a strong association with various gastric diseases, including gastritis, ulcers, and gastric cancer. Antibiotic treatment regimens have low success rates due to the rapid occurrence of resistant *H. pylori* strains, necessitating the development of novel anti-*H. pylori* strategies. Here, we investigated the therapeutic potential of a novel peptide, Tilapia Piscidin 4 (TP4), against multidrug resistant gastric pathogen *H. pylori*, based on its *in vitro* and *in vivo* efficacy. TP4 inhibited the growth of both antibiotic-sensitive and -resistant *H. pylori* (*Cag*A^+^, *Vac*A^+^) via membrane micelle formation, which led to membrane depolarization and extravasation of cellular constituents. During colonization of gastric tissue, *H. pylori* infection maintains high T regulatorysubsets and a low Th17/Treg ratio, and results in expression of both pro- and anti-inflammatory cytokines. Treatment with TP4 suppressed Treg subset populations and pro- and anti-inflammatory cytokines. TP4 restored the Th17/Treg balance, which resulted in early clearance of *H. pylori* density and recovery of gastric morphology. Toxicity studies demonstrated that TP4 treatment has no adverse effects in mice or rabbits. The results of this study indicate that TP4 may be an effective and safe monotherapeutic agent for the treatment of multidrug resistant *H. pylori* infections.

## INTRODUCTION

*Helicobacter pylori* are microaerophilic, Gram-negative bacillus bacteria that colonize the gastric mucosa. It is recognized as the major aetiological factor in gastritis, gastric ulcers, duodenal ulcers, and gastric cancer [1-5]. Prolonged *H. pylori* infection and inflammation due to bacterial virulence and host genetic factors are important risk factors for several gastro-duodenal diseases, including gastric malignancy and peptic ulcer diseases [6, 7]. Recent studies claim that *H. pylori* infection may also lead to non-gastric diseases, such as idiopathic Parkinsonism and non-arteric optic ischemic neuropathy [8-10]. Meta-analysis has revealed that the efficacy of antibiotic regimens, including clarithromycin, have declined over time, due to the increase in resistance [11, 12]. *H. pylori* infection is increasingly challenging to cure because of the dominance of resistance to antibiotics, in particular metronidazole (MTZ), which is a key component of the triple-therapy regimen [13]. The protracted nature of *H. pylori* colonization and failure of treatment necessitated that treatment strategies change from mono- to triple-, and ultimately quadruple- and rescue-therapies [14]. Long-term treatments, combined with high doses of antibiotics and patient noncompliance, have led to the development of antibiotic resistance in *H. pylori*, and the spread of resistant strains. As a consequence, there is an alarming need for new anti-*H. pylori* treatments with both superior therapeutic efficacy and negligible adverse effects.

Cationic gene-encoded host defense peptides (HDP) are nature's most diverse and lavish class of antibiotics. Most higher organisms harness these peptides as part of their innate immune systems. A subclass of HDP, known as antimicrobial peptides (AMP), exert direct antimicrobial activity by damaging bacterial membrane integrity and/or by translocation through membranes. AMPs can also inhibit intrabacterial processes, such as cell wall synthesis, DNA/RNA/protein synthesis, and even cell division [15]. Thus, AMPs with low molecular weights, composed of about 12 to 60 amino acids, are potential novel antimicrobial agents against human and animal pathogens. Recently, a much larger AMP, lactoferricin, has been identified, indicative of greater AMP diversity [16-18]. AMPs also exhibit a wide range of diversity in sequence, structure, charge, and abundance of specific amino acids [19]. AMPs may exert potent and rapid activity against a broad range of bacteria, viruses, fungi, and protozoa. This is achieved through cell lysis via direct binding, intracellular processes, neutralization of endotoxins, and wound healing [15, 20, 21]. More significantly, these molecules are of particular interest for potential therapeutic use because of their ability to kill multidrug resistant microorganisms [22]. Many AMPs are undergoing preclinical and clinical trials. Pexiganan (MSI-78), a broad-spectrum AMP, was the first to undergo commercial development [23]. Omiganan (MBI-226), another commercial AMP, is a synthetic analogue of indolicidin, which has broad antibacterial and antifungal activity [24].

A class of cationic AMPs called Piscidins was recently found to be expressed by fish mast cells [25]. Piscidin AMPs are made up of 21~ 44 residues, and possess an amphipathic-helical structure [26, 27]. Synthetic piscidin 2 has fungicidal activity against *Candida albicans*, *Malassezia furfur*, and *Trichosporon beigelii in vitro* [28]. In a previous study, we isolated five novel piscidins from Tilapia, and examined their antimicrobial and anti-fungal activity, revealing these compounds to be potent and promising antimicrobial agents with broad spectra of activity.

A more complete understanding of how these short amphipathic cationic AMPs bring about bacterial cell death and host immunomodulation is needed for further optimization and development for clinical applications. We previously demonstrated the latent effect of Tilapia piscidins on clinically-important pathogens [29]. Here, we report that four representative Tilapia piscidin peptides exhibit antibacterial activities against *H. pylori in vitro*. Tilapia Piscidin 4 (referred to hereafter as TP4) demonstrated the most potent activity, and was active against a broad spectrum of strains, while being remarkably stable at low pH. We further report that TP4 has multiple modes of action, including direct membrane disruption and interference of intracellular processes, as well as counterbalancing the host immune system. We previously reported that TP4 exhibits low toxicity against human and mouse cell lines, and low hemolytic activity [29]. In this study, we show that: (i) TP4 exerts its antimicrobial effects against bacteria via disruption of membrane potential; (ii) the vulnerability of clinical isolates to TP4 is not associated with preexisting resistance to antibiotics; (iii) TP4 reduces *H. pylori* infection by specifically inducing host adaptive immune responses against persistent colonization in gastric tissue; and (iv) TP4 acts in a synergistic manner with conventional antibiotics. Collectively, our results suggest TP4 as a promising agent for use against multidrug resistant *H. pylori*.

## RESULTS

### Determination of MIC and MBC for Tilapia Piscidins 1~5 against *H. pylori*

Our previous study demonstrated that the Tilapia Piscidins are potent against Gram negative and Gram positive pathogens [29]. In this current study we investigated the minimal inhibitory concentrations (MICs) of five Tilapia Piscidins against various *H. pylori* strains, including antibiotic resistant clinical isolate CI-HP028. The MIC values are presented in Table 1. With the exception of TP2, all peptides (TP1, TP3, TP4, and TP5) inhibited the growth of *H. pylori*. The peptide with the strongest efficacy was TP4, and so it was selected for in-depth characterization (TP2 was used as a control peptide due to its lack of anti-*H. pylori* activity). The data in Table 1 indicate that all four strains of *H. pylori* are sensitive to TP4 (MIC- 1.5-3 μg mL-1), irrespective of whether the strain is sensitive or resistant to antibiotics.

**Table 1:** Antimicrobial activities of Nile Tilapia Piscidin 1~5 peptides against laboratory and antibiotic resistant clinical isolate *H. pylori* strains

	MIC (µg mL-1)^a^				
Tilapia Piscidin designation	Sequences^29^	*H. pylori* strains			
		43504	700392	43629	CI-HC-028^b^
**TP1**	FDWDSVLKGVEGFVRGYF	> 12	> 12	> 12	> 12
**TP2**	GECIWDAIFHGAKHFLHRLVNP	NE^c^	NE	NE	NE
**TP3**	FIHHIIGGLFSVGKHIHSLIHGH	8-12	8-12	8-12	8-12
**TP4**	FIHHIIGGLFSAGKAIHRLIRRRRR	1.5-3*	1.5-3*	1.5-3*	I.5-3*
**TP5**	QLQGKQVSGEVVQKVLQELIQSVAKP	>12	>12	>12	>12

a The minimum inhibitory concentration

b CI-HC-028: Clinical isolate

c NE: No effect

* All *H. pylori* strains exhibit significant sensitivity to TP4 (p < .01)

### Dose and time killing curves

**Figure 1:**
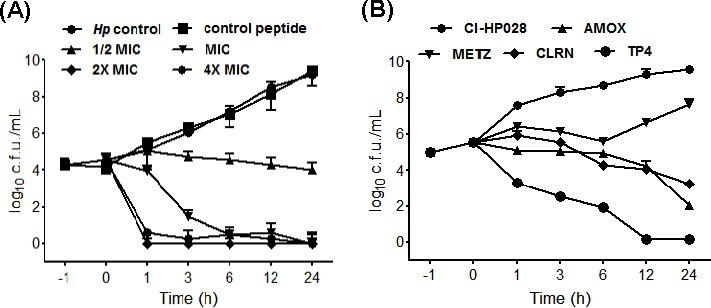
Dose- and time-dependent killing kinetics of TP4 against *H. pylori* (**A**) Approximately, 2 × 10^5^ cells were incubated for 1 h, and then incubated with the indicated concentrations (1/4, 1/2, 1, 2, and 4 fold of MIC) of TP4. Control peptide and *H. pylori* alone were used as controls. The cultures were monitored for 24 h, and aliquots were taken at 1, 3, 6, 12, and 24 h for bacterial enumeration. The data are means ± SEM. (**B**) Time-dependent killing effects of TP4 and antibiotics on preexisting antibiotic resistant clinical isolate *H. pylori* (CI-HP028). Approximately, 1 × 10^5^ cells were incubated for 1 h, and then incubated with the MIC of TP4, amoxicillin, metronidazole, or clarithromycin (AMOX, METZ, and CLRN, respectively). The cultures were monitored for 24 h, and aliquots taken at 1, 3, 6, 12, and 24 h to determine surviving c.f.u. The data are means ± SEM

The rate of antimicrobial activity is important, as it determines whether the required concentration is maintained for sufficient time in a harsh gastric environment. Therefore, we examined dose and time kill curves against *H. pylori*. The bactericidal rate was found to be 99% and 99.9% at MIC and 2 × MIC, respectively. As shown in Figure 1A, no colonies were recovered after treatment with 1 × MIC of TP4. The time required to establish a 99 % killing rate was about 180 minutes. Incubation with 2 or 4 × MIC increased the killing rate. Taken together, these data indicate that the efficacy of TP4 is reliant on both dose and duration. These data also suggest that TP4 binds to a fixed number of bacterial targets.

### TP4 antimicrobial activity is independent of antibiotic resistance

We next compared the time-based bactericidal activities of TP4 and conventional antibiotics against multidrug resistant clinical isolate CI-HC-028 (certain strains are metronidazole and clarithromycin resistant) [30]. We observed that by ~6 hr, TP4 treatment had reduced *H. pylori* by >3 log, while, of the antibiotics, only amoxicillin caused 90% killing within 24 hr (see Figure 1B). Metronidazole caused an initial decrease in cell count, but gradually lost activity; clarithromycin consistently reduced c.f.u., but the time taken to 90% reduction was >24 hr. Thus, there is no correlation between multidrug resistant (MDR) phenotype and vulnerability to TP4. Therefore, TP4 is superior to conventional antibiotics against MDR bacterial strains.

### Development of drug resistance

**Figure 2:**
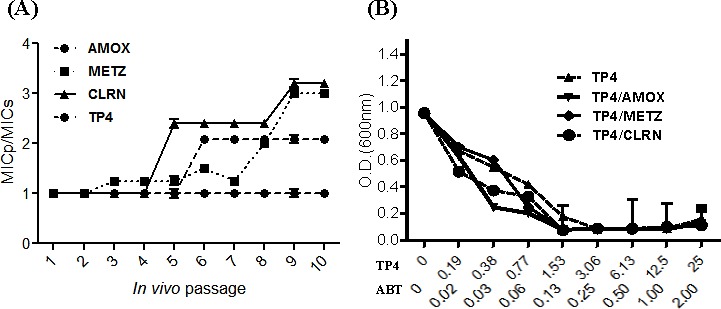
*In vitro* passaging with sub-inhibitory concentrations of TP4 does not induce resistance in *H. pylori* (**A**) *H. pylori* were passaged *in vitro* in the presence of sub-lethal doses of TP4 or the indicated antibiotic, and then infected into mice for *in vivo* passage. Animals were sacrificed at each passage, and the MIC of TP4, amoxicillin, metronidazole, and clarithromycin were recorded for 10 serial passages. Passage points in the curves are means ± SEM. (MICs: standard MIC value; MICp: passage-induced MIC). (**B**) *In vitro* dose-OD fall kinetics were examined for TP4 alone and in combination with amoxicillin, metronidazole, or clarithromycin. Synergism was observed for all peptide-drug combinations against metronidazole- and clarithromycin-resistant *H. pylori* strains. The data shown are means ± SEM of two independent experiments

Inappropriate antibiotic use and poor compliance with therapeutic regimens is a major driver of drug resistance [31]. Here, we established a model for simulating conditions of drug resistance emergence: bacteria were exposed to sub-inhibitory doses of amoxicillin, metronidazole, clarithromycin, or peptide TP4 *in vitro*. C3H/HeN mice were infected with the resulting inocula, and colonized mouse gastric tissue was subsequently harvested for evaluation of drug susceptibility (drug resistance index MICp/MICs). This process was repeated for over 10 passages (see Figure 2A). The resistance index for amoxicillin increased slightly after passage 6, and remained constant thereafter. For clarithromycin and metronidazole, the resistance indices (MIC) gradually increased from passage 3 to the last passage. However, the resistance index for TP4 did not change during the course of our experiment. These findings suggest that bacteria may not readily develop resistance to this AMP.

### TP4 exhibits synergistic activity with antibiotics against resistant *H. pylori*

Combination therapy often enhances antibiotic efficacy and mitigates the frequency at which drug resistance emerges [32]. To determine the suitability of TP4 for combination therapy, we examined *in vitro* dose-OD fall kinetics of TP4 in combination with AMOX, CLRN, or METZ antibiotics; these three antibiotics are traditionally used as the first line of defense against *H. pylori* [33]. We report that TP4 has a significant synergistic effect, reducing the MIC of amoxicillin by one-fourth and the MIC of metronidazole and clarithromycin by one-half (see Figure 2B).

### *In vitro* antimicrobial mechanism

#### NPN fluorescence and transmission electron microscopy studies reveal that TP4 disrupts the *H. pylori* membrane via micellization

**Figure 3:**
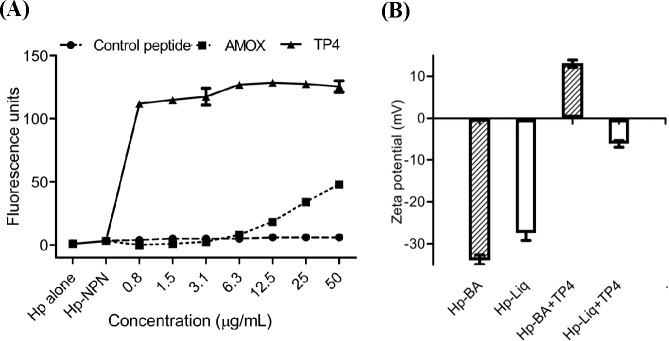
TP4 induces membrane permeation of *H. pylori* TP4 rapidly permeabilizes the *H. pylori* outer membrane in a dose-dependent manner. Membrane permeabilization was examined by measuring fluorescence due to uptake of 1-*N*-phenyl-naphthylamine (NPN). Approximately 5 × 10^6^ *H*. *pylori* (Hp) cells were cultured in the presence of TP4 or amoxicillin for 6 h. Bacterial cells alone, or cells incubated with NPN and control peptide, were used as negative controls. NPN fluorescence intensity was recorded as a correlate of membrane permeation. (**B**) Zeta-potential (ζ) of *H. pylori* in the presence/absence of TP4. Striped bars: *H. pylori* grown in blood agar plates; white bar: liquid culture. The data shown are means ± SEM of two independent experiments

Cationic AMPs have been shown to target and disrupt bacterial membranes and/or bind to internal constituents in a manner that may interrupt biomolecule synthesis [34]. Thus, we examined whether TP4 induces permeation and disruption of the *H. pylori* cell membrane. We assessed membrane integrity using 1-N-phenylnaphthylamine (NPN) [35]. Generally, NPN is blocked by intact bacterial cell membranes. However, when outer membrane assembly is disrupted, NPN easily passes through the barrier into the hydrophobic interior of the outer membrane, resulting in a rapid increase in fluorescence. We thus examined NPN fluorescence intensity following TP4 treatment (see Figure 3A). TP4 increased fluorescence at sub-MIC values, indicating that TP4 effectively permeabilizes membranes. However, AMOX treatment also caused a slight increase in fluorescence intensity at higher doses. To confirm that TP4 increases the porousness of the cell membrane, we analyzed the surface charge (zeta potential (ζ)/mV) of bacterial cultures (see Figure 3B) [36]. *H. pylor*i inocula from blood agar and liquid cultures displayed zeta potentials of −33.83 and −27.47 mV, respectively. Addition of TP4 at the MIC to blood agar or liquid inocula of *H. pylori* increased the zeta potential to +13.03 and −6.18 mV, respectively. These data strongly suggest that TP4 has affinity with the *H. pylori* surface membrane, and thereby lyses the membrane.

#### Transmission electron micrography (TEM) analysis of the effect of TP4 on *H. pylori* membrane morphology

**Figure 4:**
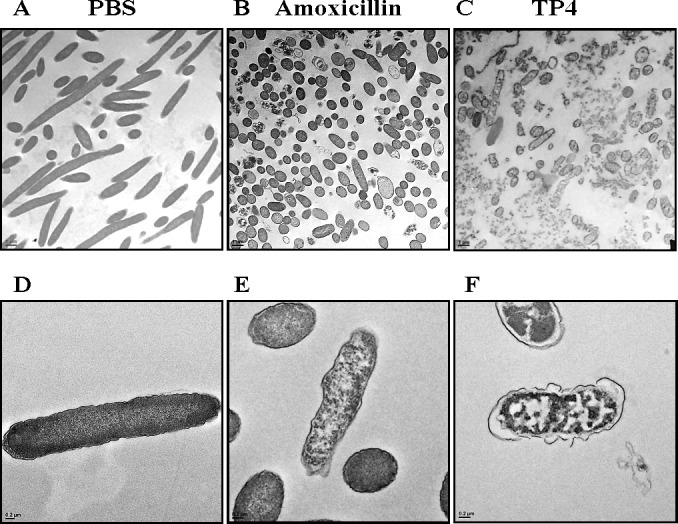
Electron micrographs reveal that TP4 causes membrane disruption in *H. pylori* via micellization Approximately 6 × 10^7^ *H. pylori* cells were exposed to PBS (**A**, **D**), 1x MIC amoxicillin (**B**, **E**), or 1x MIC TP4 peptide (**C**, **F**) for 2 hr. Next, thin sections were examined under a transmission electron microscope (TEM) at low (1,100X; A-C) or high (5,500X; D-F) magnification. (A) Normal spiral- or comma-shaped rod morphology of *H. pylori* cells and intact cell membranes were observed in the control sample. (**B**) Amoxicillin-treated cells exhibited a shift from long spiral to coccoid forms, but no visible lysis of membrane structures. (**C**) TP4 treatment resulted in the appearance of abundant ‘ghost’ cells, and major cell death due to membrane lysis. (**D**) Control cells exhibited curved and intact morphology. (**E**) Amoxicillin-treated cells retained intact membranes, but the periplasmic space was swollen in some locations. (**F**) TP4 treatment induced the formation of electron-dense structures within cells and the extracellular media

Next, we used TEM to inspect the ultrastructure of cells following treatment with TP4 (see Figure 4) and amoxicillin. Low and high-magnification images of control *H. pylori* cells reveal long, regular spiral structures, and few circular coccoid cells with intact cell membranes (see Figure 4A, 4D). Upon early exposure to amoxicillin, however, *H. pylori* appeared as “ghost” cells, with loose outer membranes. Interestingly, amoxicillin treatment did not alter the coccoid forms [37, 38] (see Figure 4B, 4E), while TP4 treatment resulted in substantial disruption of bacterial cell membranes through micellization (see Figures 4C, 4F). TP4 treatment also resulted in an abundance of readily observable electron-dense structures. The effects of TP4 detectable shortly after exposure (120 minutes) are consistent with the observed NPN membrane permeabilization time (see Figure 3A) and the time kill assay data (see Figure 1A).

**Figure 5:**
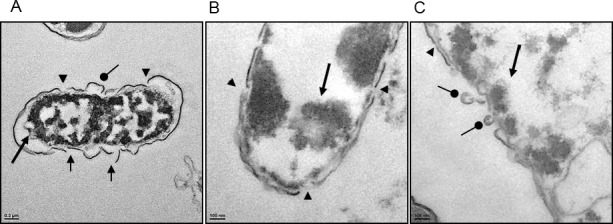
High magnification electron micrographs confirm that TP4 induces micellization of the *H. pylori* membrane (**A**) Electron micrographs of *H. pylori* at 7,000× magnification reveal characteristic outer membrane disruption. (**B** and **C**) Micrographs at 15,000× magnification; short round arrowheads indicate micelle formation sites, arrow heads indicate nick regions where micellization occurs, short arrows indicate missing membrane sections, and long arrows indicate electron dense aggregates inside cells (Scale bars: (**A**) 200 nm; (**B**, **C**) 100 nm)

At higher magnifications (X7000-15000), we observed extensive membrane perturbation of *H. pylori* following exposure to TP4 (Figure 5A-5C)); specifically, we observed protruding micelles and membrane sloughing (see Figure 5A). In addition, we observed missing membrane sections (see Figure 5B) and nicks indicative of micellization, as well as detachment of bacterial-TP4 micelles (see Figure 5C). Outer membrane destruction was evidenced by the formation of micelles, which in turn resulted in membrane depolarization and leakage. As expected, the morphology is consistent with the observed zeta potential (see Figure 3B). Taken together, these data indicate that internalized TP4 disrupts intracellular components, thereby causing *H. pylori* cell death through induction of membrane micellization [39-41]. A proposed mechanism of action representing TP4 action and its significant events are depicted in Figure 12.

#### Efficacy of TP4 *in vivo* against *H. pylori* in a mouse model of infection

**Figure 6:**
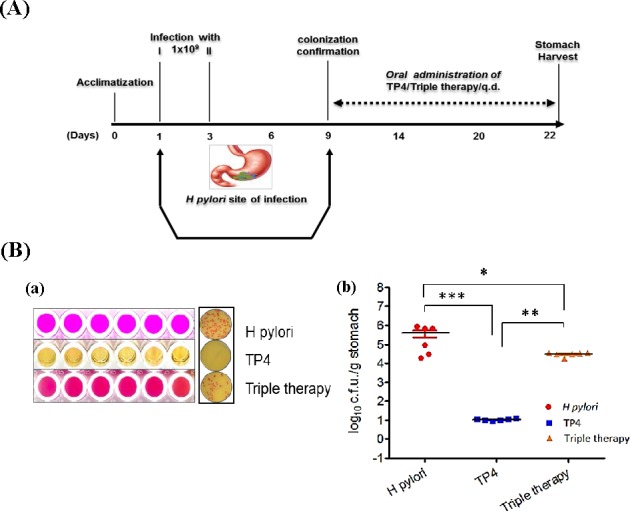
Anti-*H. pylori* efficacy *in vivo* (**A**) Scheme depicting infection and treatment of C3H/HeN mice to investigate *in vivo* efficacy of TP4 against *H. pylori*. All mice were orally infected with approximately 1×10^9^ c.f.u. of *H. pylori* on days 1 and 3. On day 9, mice were euthanized and stomach tissue was harvested. Animals were divided into three groups: (i) *H. pylori* infection alone; (ii) infection and treatment with TP4 treatment; and (iii) infection and treatment with triple therapy (clinical antibiotics: amoxicillin, metronidazole, and clarithromycin). Groups (ii) and (iii) were given orogastric doses of 10X MIC of TP4/Triple therapy complex for two weeks. After completion of treatment, mice were euthanized, and stomach, spleen, and blood were harvested for use in various cytological and biochemical assays. (**B**) a) Biochemical urease test for *H. pylori* identification in gastric tissue lysate. From top: untreated mice, mice treated with TP4, and mice treated with triple therapy. Pink spots indicate *H. pylori* infection. Right: cultured selective media plates (Fig. 6(B) a). b) Enumeration of bacterial burden in the stomachs of *H. pylori*-infected mice, and infected mice treated with TP4 or triple therapy (n = 6 per group). Bars represent median values with **p < 0.05, *** p < 0.001 (Fig. 6(B) b)

The *in vitro* data described above suggest that TP4 has strong activity against *H. pylori*. We proceeded to evaluate the *in vivo* therapeutic efficacy and immunomodulatory properties of TP4 against *H. pylori*. The experimental plan to establish *in vivo* infection is represented schematically in Figure 6A. At 1 wk post infection, mice were divided into 3 groups (n = 6) and treated with PBS, TP4, or PPI-Triple therapy.

After treatment, rapid urease tests were performed to confirm that *H. pylori* was present in the gastric tissue of untreated mice or mice treated with triple therapy, but not of mice treated with TP4 (see Figure 6B (a)). Furthermore, ; 7 × 10^5^ c.f.u./g of *H. pylori* was detected in the stomach tissue of mice in the untreated group; this was significantly reduced to 5 × 10^4^ c.f.u./g in mice treated with triple therapy (p=0. 0187) (Figure 6B(b)). Several methods were unable to detect bacteria in the gastric tissue of mice treated with TP4, but the probability of bacterial presence was set as 1 for statistical comparison. As compared with the triple therapy group, TP4 caused a significant reduction of bacterial burden (p = 0.00152). This confirms that TP4 treatment significantly cleared the *H. pylori* burden from the stomachs of infected mice.

#### Effects of TP4 on gene and protein expression of *H. pylori* virulence factors

**Figure 7:**
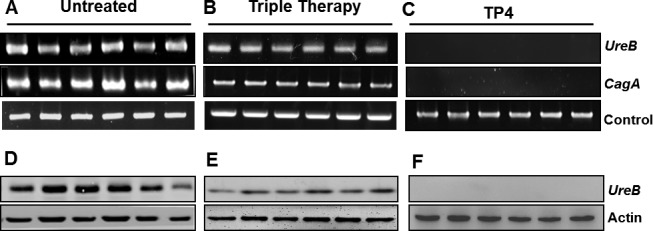
Effects of TP4 on gene and protein expression of *H. pylori* virulence factors Gene and protein expression of Type IV Secretory CagA and Urease B were examined. Gastric tissue genomic DNA was isolated from infected mice that had been (**A**) untreated, or treated with (**B**) Triple therapy or (**C**) TP4 (n=6). Western blot analyses of urease toxin protein in gastric tissue lysates from the indicated groups are shown in panels D-F

There is evidence for differing degrees of virulence between *H. pylori* strains, arising from the presence or absence of virulence factors [42, 43]. PCR tools used to amplify certain putative virulence markers (the *cagA* and *ureB* genes) provide a sensitive means of detecting *H. pylori* colonization in the stomach (see Figure 7A-7C). Infected mice were found to be positive for both *ureB* and *cagA*, confirming colonization at the molecular level. Treatment with triple therapy failed to eradicate *H. pylori*, but decreased band intensity. However, the TP4-treated group tested negative for both the ureB and cagA genes. We suggest that PCR for *ureB* and *cagA* may be suitable for use as a diagnostic tool after therapy [44]. We proceeded to examine gastric lysates for *H. pylori* virulence protein urease B, which is pivotal for colonization in the acidic environment of the stomach. Urease B protein was detected at high levels in the infected group, while triple therapy decreased protein expression, and TP4 treatment abolished detectable protein (see Figure 7D-7E). These data, together with the membrane and surface charge disruption findings (see Figure 3A and 3B), suggest that the *H. pylori* virulence factors *Cag A* and *Urease* are released in outer membrane vesicles, which are engendered from the bacterial membrane and possess the same composition and surface charge as the parent membrane. In summary, TP4 clears extracellular bacteria and their outer membrane vesicles.

#### Histological examination and special staining analysis

**Figure 8:**
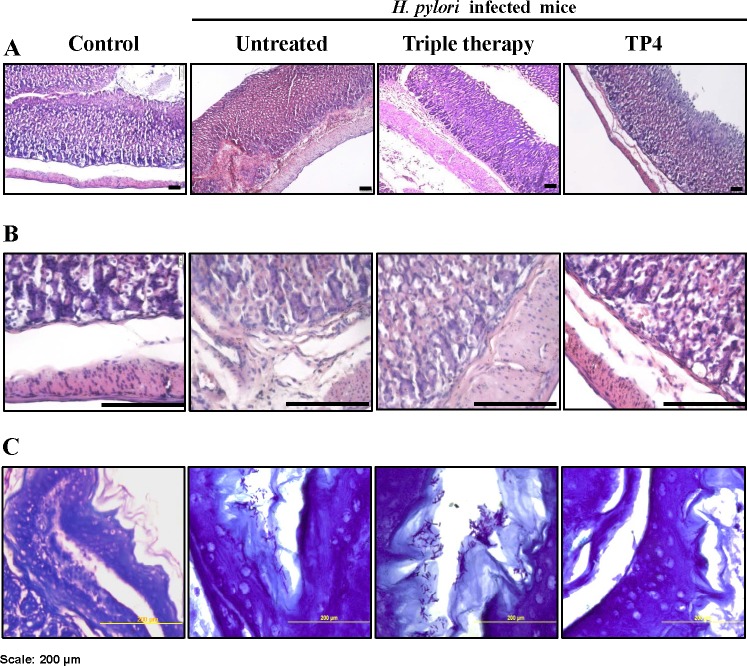
Histopathology analysis of *H. pylori-*infected mice without treatment or after treatment with PPI-triple therapy antibiotics or TP4 Sections were stained with HE to score gastric inflammation during infection and after therapy (**A**). Images at higher magnification to view immune cell infiltration are shown in (**B**). (**C**) *H. pylori*-specific special stained slides. Scale bar: 200 μm

Histological examination of gastric tissue sections stained with hematoxylin and eosin revealed severe inflammation in infected mice (see Figure 8A, 8B). Furthermore, the ulcer crater and muscularis mucosae layer were heavily infiltrated by inflammatory cells. By intruding upon the chief and parietal cell regions, these inflammatory cells resulted in superficial damage to the surface epithelium, which is a known response to severe *H. pylori* biased-host immune responses upon colonization. Triple therapy did not reduce inflammation. However, treatment with TP4 significantly reduced *H. pylori*-induced inflammation, and cleared immune cell infiltration at ulcer crater and muscularis mucosae, thereby restoring gastric tissue morphology. Special modified Giemsa staining was performed to detect *H. pylori* colonization of the gastric mucosa (purple colored spirals; see Figure 8C). Untreated and triple therapy-treated mice exhibited significant *H. pylori* burdens in the gastric mucosae; treatment with TP4 monotherapy significantly cleared the *H. pylori* burden in gastric tissue. Thus, TP4 treatment considerably reduces the gastric bacterial burden and restores normal gastric morphology.

### Mechanism of host immunomodulation

#### Effects of TP4 on splenic CD4^+^Foxp3, Th17 T-Cells, and TH17/Treg ratio dynamics in a mouse model of *H. pylori* infection

**Figure 9:**
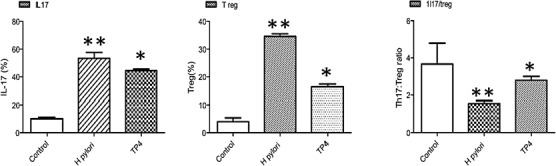
Immunomodulatory effects of TP4 in *H. pylori*-infected mice Effects of TP4 treatment on splenic T cell subsets induced in *H. pylori*-infected mice. Splenic T subset populations were quantified using fluorescent antibodies against inflammatory and anti-inflammatory T cells

It was previously reported that *H. pylori* infection induces regulatory T cells (Tregs), which leads to *H. pylori* persistence [45]. Foxp3+ Tregs and interleukin (IL)-17+ helper T (Th17) cells have been implicated in host immunity to *H. pylori*, particularly at gastric mucosal surfaces. *H. pylori* persistence may depend on the Th17/Treg balance, as arbitrated by *H. pylori*-communicating antigen presenting cells [46]. *H. pylori* evades immune clearance by tuning the host immune response to sustain low Th17/Treg. Flow cytometry was used to demonstrate that TP4 strongly inhibits Treg cells and moderately suppresses Th17 cells, which increases the Th17/Treg ratio in splenic T subsets (see Figure 9). This suppression of *H. pylori*-biased host immune responses upon treatment with TP4 dynamically clears *H. pylori* colonization and reduced gastric inflammation.

#### Expression of gastric cytokines and T cell markers

**Figure 10:**
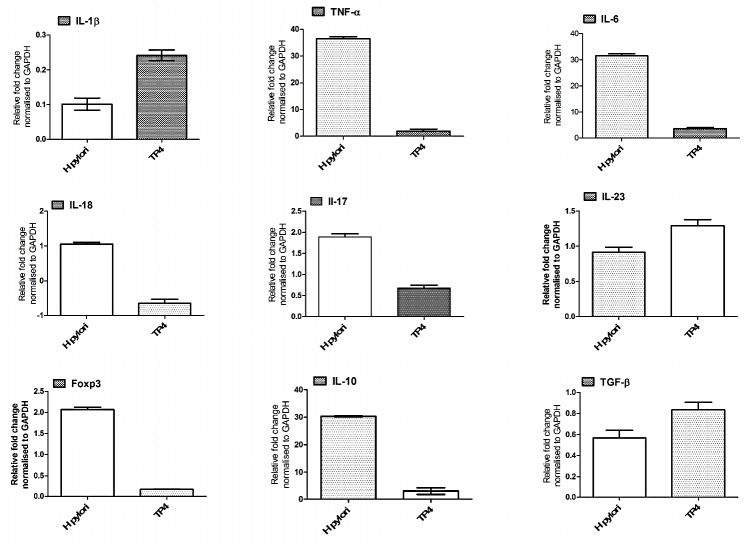
Effects of TP4 on expression of different inflammatory and anti-inflammatory genes in H *pylori-*infected mice. Gastric tissue mRNA was isolated from untreated and treated mice, and real-time PCR was used to measure mRNA expression for the indicated genes. Data are representative means ± SEM.

We subsequently used real time-PCR to analyze the expression of genes involved in host immune responses in the gastric tissues of infected mice treated with or without TP4. We discovered that gene expression of certain cytokines (Tumor necrosis factor-α, IL-6, IL18, IL-10, IL-17, Il-23, and TGF-β) and the T-cell marker FOXp3 were profoundly increased during *H. pylori* infection (see Figure 10). Of these cytokines, IL-10, IL-18, and Il-17 are known to influence tenacious *H. pylori* colonization and gastric inflammation. TP4 treatment resulted in suppression of all of these genes, with the exceptions of Il-23 and TGF-β. A decrease in IL-10 *in vivo* results in a significant reduction of Treg cells, and restores the host-Th17/Treg balance. Further, moderate inhibition of IL-17 facilitates the repair of inflamed gastric tissue (evident in the histopathology data shown in Figure 8).

#### *In vivo* evaluation of TP4 toxicity

**Acute oral toxicity**: a single dose of 125 mg/kg was orally administered to C3H/HeN mice (n=6), and animal morbidity or mortality was observed for 24 hours. Mice did not exhibit any abnormal clinical signs, and no abnormalities in vital organs were detected [47].

**Sub-acute oral toxicity**: Three-fold therapeutic doses were orally administered to mice for 14 days. The animals were observed for any clinical signs of morbidity or mortality from days 1 to 28. TP4 did not induce clinical complications in mice, and no abnormalities were observed on day 28 after euthanasia (n=6) [48].

#### Eye irritation test in rabbits

**Figure 11:**
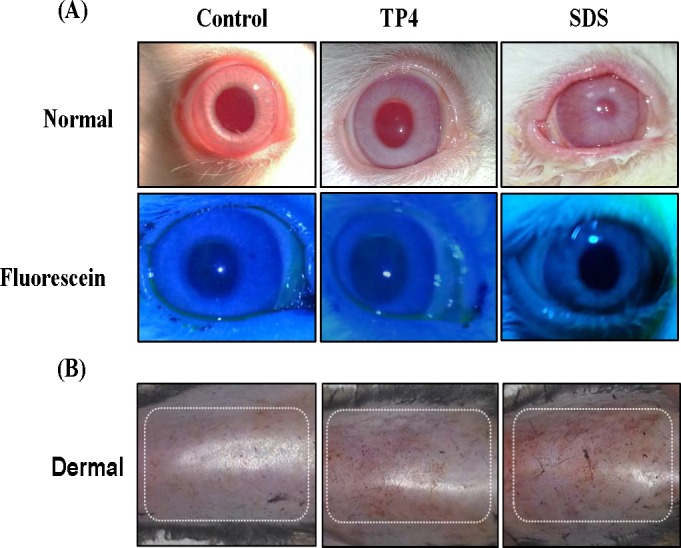
TP4 toxicity safety evaluations in New Zealand albino rabbits and C3H/HeN mice (**A**) Eye irritation test: Ocular appearances of controls, and rabbits treated with TP4 or SDS for 7 days. The animals were observed daily, and no ocular lesions were detected in the control or TP4-treated rabbits. The positive control SDS caused severe lacrimation, swelling of the eyelids, and elevated blood vessels in conjunctivae. The lower panel shows fluorescein stain images. (**B**) Dermal toxicity: appearances of the skin of mice treated with TP4 at 48 hr post-exposure. No obvious lesions were observed in mice treated with 125 mg/kg TP4 or 20% SDS after residue removal. TP4 exposure was extended for 7 days, and animals were observed daily until day 14; no clumps or lesions were observed in TP4-treated mice at the end of the study (n=12 per group).

A summary of the eye irritation clinical signs observed during TP4 treatment is provided in Table 2 and are shown in Figure 11A [49]. Rabbits treated with TP4 exhibited very mild corneal opacity (clouding of the cornea) on day 5 and 7, which was insignificant compared to the eyes of positive controls (rabbits treated with SDS). No rabbits treated with TP4 or SDS exhibited abnormalities in the iris (lesions/tear/inflammation). TP4-treated rabbits presented with slight conjunctivae redness (palpebral/bulbar conjunctivae) on day 5, which was not apparent at subsequent days. TP4 also caused slight chemosis (swelling of the eye lids/nictitating membranes) of the lower lids on days 3, 5, and 7, but this was minor compared to that observed in positive control animals; moreover, the chemosis of TP4-treated animals disappeared during the recovery period. Animals were sacrificed on day 14, and no abnormalities in the vital organs, or lesions or clumps in the eyes, were observed in TP4-treated rabbits.

**Table 2:** Grades of signs and symptoms of eye irritation in response to peptide TP4 in rabbits

Ocular signs and grading					
Treatment groups	Observation	Corneal opacity	Abnormality iris	Conjunctivae Redness	Chemosis
TP4	Day 1	0	0	0	0
	Day 3	0	0	0	0.1
	Day 5	0.1	0	0.5	0.1
	Day 7	0.3	0	0	0.1
Recovery	Day 14	0	0	0	0
SDS	Day 1	1	0	1	1.5
	Day 3	1	0	1	1.5
	Day 5	1	0	1	2
	Day 7	1	0	1	2
Recovery	Day 14	0.5	0	0.5	1.5

* Data are graded based on OECD 405, guidelines for eye irritation test.

**Dermal toxicity**: Dermal exposure of C3H/HeN to TP4 did not result in mortality or damaging effects at any point during the 14-day observation period (see Figure 11B). Furthermore, the body weight of treated animals was normal until the last day of observation. TP4 treatment did not induce lesions or irritation during the test period, and no gross behavioral changes were observed. Hair growth was also found to be normal, and necropsies did not reveal any gross abnormalities in organ structure or architecture [50].

## DISCUSSION

The worldwide distribution of “pandrug” and “multidrug” resistant pathogens has severely compromised the efficacy of our antibiotics, and dramatically increased the occurrence of therapeutic failure [51]. Triple and quadruple therapies have had some success in eradicating resistant infections, but these also result in adverse effects, such as diarrhea and soft stool [52]. Excessive use of antibiotics is also a major cause of imbalances in the normal flora of the gut region. Moreover, *H. pylori* is currently developing resistance to many clinically important antibiotics, most likely because of the spread of efflux pumps [54] and changes in bacterial cell membrane composition which prevent these agents from diffusing efficiently across the cell membrane [53]. One potential alternative to antibiotics is AMPs, the efficacy of which is strongly hinted at by their pervasive presence among eukaryotes [55]. Thus, here we evaluated the antimicrobial activity of TP4 against antibiotic susceptible and resistant *H. pylori* strains. TP4 is of the Tilapia Piscidin class of AMPs, with potential activity against many pathogenic and clinically challenging species, but reportedly not against *Helicobacter* species [29]. Much like the AMP Epinecidin-1, TP4 rapidly kills both Gram negative and positive bacteria [56]. Here, we provide both *in vitro* and *in vivo* experimental confirmation that TP4 possesses potent antimicrobial activity against *H. pylori*, and report that TP4 permeates the bacterial membrane and balances the host immune response in a mouse model of *H. pylori* infection.

Initially, we evaluated the efficacy of TP4 against standard laboratory strains. However, the key challenge facing antibiotic development is the treatment of infections caused by multidrug-resistant strains. MIC-based susceptibility testing was used to show that TP4 is equally efficacious against both antibiotic-sensitive and-resistant gastric pathogen *H. pylori* strains. Analysis of time-dependent kill kinetics revealed marked growth inhibition as early as 120 minutes post exposure for different *H. pylori* strains. The activity of TP4 was found to be independent of preexisting antibiotic resistance. These data indicate that TP4 alone or in combination with a commonly-used antibiotic may reduce therapy duration, and be suitable for use against a wide range of antibiotic-resistant strains.

**Figure 12:**
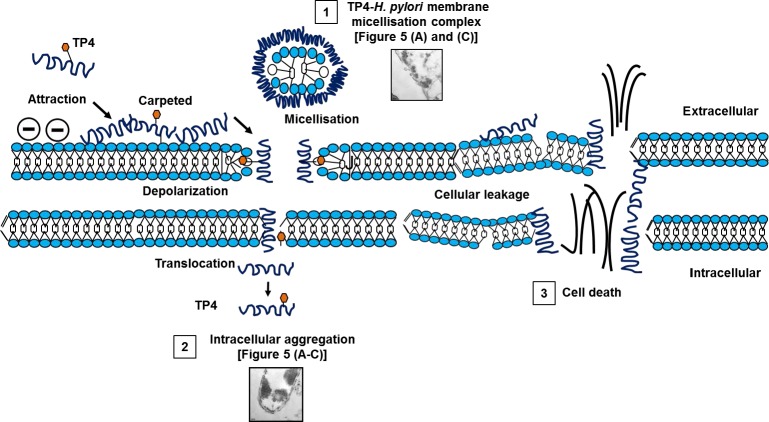
Proposed mechanism of action of TP4 against *H pylori* 1) Membrane micellization. 2) Translocation and intracellular aggregation. 3) Cellular leakage of essential respiratory ions causes osmotic imbalance, and subsequent cell death.

To elucidate the *in vitro* mode of TP4 action, we performed NPN membrane leakage assays, measured zeta-potential, and examined bacteria under transmission electron microscopy. We report that TP4 has strong affinity with the negatively-charged molecular structures of the membrane and induces membrane micellization, as evidenced by electron micrographs revealing lost membrane sections (proposed mechanism of action; Figure 12 (1)). Further analysis demonstrated that the loss of membrane integrity is accompanied by marked membrane depolarization, based on both NPN fluorescence and measurement of zeta potential (see Figure 12 (2 and 3)).

Recent clinical trials using AMPs which interfere with membrane integrity and function raise the possibility that TP4 (or other AMPs) may also be valuable for treating infections, including those caused by MDR bacteria [57]. Even in situations where a given AMP does not exhibit any improvement in therapeutic ratio as compared with antibiotics, combination therapy may hold promise for clinical applications, as suggested by the synergistic effect of TP4 with amoxicillin, clarithromycin, and metronidazole against antibiotic-resistant strains.

We also demonstrated that monotherapeutic treatment of animals with TP4 significantly reduced *H. pylori* colonization (see Figure 6(b)). This suggests that TP4 is stable and active in the harsh acidic and proteolytic environment of the stomach, despite reports that such conditions are commonly associated with reduced bioavailability of some antibiotics. Our preliminary studies on pH stability show that TP4 is active under physiological acidic conditions, due to maintenance of its high positive net charge by histidine, lysine, and arginine [58-60]. Furthermore, TP4 must be able to penetrate the mucous layer to reach the colonized mucosal surface; however, the mechanism by which this is accomplished is not clear at present.

In addition, we have shown here that TP4 selectively suppresses *H. pylori*-induced host immune responses (see Figure 9, 10). *H. pylori* infection induces Treg cells in the gastric tissue to suppress effector T cell development [61, 62]. Such significant Treg polarization aids in the persistence of *H. pylori* colonization. It was recently reported that vaccine efficiency can be improved using a peptide inhibitor of FOXP3, which impairs Regulatory T cell development. Similarly, peptide TP4 selectively suppresses the cytokines and T cell markers responsible for Treg cell development, thereby balancing the host immune response for clearance of persistently colonized *H. pylori* in gastric tissue. Finally, we performed toxicity and safety evaluations to establish that TP4 does not cause dermal irritation or hypersensitivity in mice or rabbits.

## CONCLUSIONS

1. We have demonstrated that TP4 exerts potent antimicrobial activity *in vitro* and *in vivo* against *H. pylori* strains (an MDR clinical isolate and the ATCC 43504 strain).
2. Treatment with TP4 significantly reduced *H. pylori* in a mouse model of infection, as compared to untreated controls and mice treated with conventional antibiotics.
3. High doses of TP4 did not cause any toxic effects in mice or rabbits.
4. TP4 selectively suppressed the *H. pylori*-induced immune response by altering the expression of immune-responsive genes. Collectively, these results support the potential development of TP4 as a novel class of anti-*H. pylori* therapeutics. These experimental findings lend further support for future clinical development of TP4 as a candidate drug, and possible future suitability for use against human gastric diseases in pre-clinical trials.

## MATERIALS AND METHODS

### *H. pylori* strains and growth conditions

*H. pylori* strains, American type cell cultures (ATCC) 43504, 51653, and 700392, and a clinical isolate CI-HC-028 (provided by Dr. Ming-Shiung Wu, Department of Internal Medicine, Gastrointestinal Hepatobilary Division, National Taiwan University Hospital, Taiwan) were used to determine the antimicrobial efficacy of TP4. Strains were cryopreserved at −80°C in brain heart infusion broth supplemented with 20% FBS. Bacteria were cultured under microaerobic conditions, as previously described [63]. Briefly, bacteria were spread on brain heart infusion (BHI) agar plates supplemented with 10% (v/v) sheep blood, and plates were incubated at 37°C for 24 hr under a microaerobic atmosphere (Anaeropack-Anaero, Mitsubishi, Japan).

### Synthesis of the antimicrobial peptide TP4

Tilapia piscidin 4 was obtained from Nile tilapia (*Oreochromis niloticus*). The peptide possessed the sequence FIHHIIGGLFSAGKAIHRLIRRRRR, and the C-terminus was amidated. The peptide was synthesized by GL Biochem (Shanghai, China) using a solid-phase procedure of Fmoc chemistry [29, 64]. Crude peptides were extracted, lyophilized, and purified by reverse-phase high-performance liquid chromatography (HPLC). The molecular masses and purity of the fractionated peptide were verified by mass spectroscopy and HPLC, respectively. The obtained synthetic peptide was >95% pure, and the peptide was freshly reconstituted in PBS to generate working stocks prior to each experiment.

### *In vitro* anti-*H. pylori* assessment

Frozen cultures were streaked onto BHI agar with 10% sheep blood, and incubated for 24 hr under microaerophilic conditions [65]. *H. pylori* cells undergoing exponential growth were suspended in sterile phosphate-buffered saline (PBS), and adjusted to 0.05 OD using BHI broth; the resulting inoculum was dispensed onto a microtitre plate. Peptide TP4 was prepared at various concentrations (0.04 to 50 μg mL^−1^) using PBS. Antibiotic resistant clinical isolate CI-HC-028 and three reference strains were used for susceptibility testing. All plates were incubated under microaerophilic conditions at 37°C overnight. The MIC was defined as the lowest concentration of the drug at which there was no visible growth. For quality control and comparative analyses, we examined the susceptibility of all four *H. pylori* strains to the antibiotics amoxicillin, clarithromycin, and metronidazole (Sigma Chemical Co., St. Louis, MO).

### Dose- and time-dependent effects of TP4

TP4 peptide solutions (100 μl) at concentrations corresponding to 1/4, 1/2, 1, 2, and 4 fold the MIC were prepared and added to an equal volume of solution containing bacterial counts of approximately 10^7^ c.f.u. mL^−1^ in each well of a 96-well plate. The plates were incubated at 37°C, and samples were collected at 1, 3, 6, 12, and 24 h. Samples were diluted to the indicated amounts, and plated in triplicate onto blood agar plates; bacterial counts were determined after incubation under microaerobic conditions at 37°C for 24 hr. The results were expressed as mean log (c.f.u. mL^−1^) with standard error.

### Induction of drug resistance

The drug resistance of bacteria to TP4 and clinical antibiotics was determined by in vitro culturing of *H. pylori* in sub-inhibitory concentrations of TP4 and antibiotics, followed by repeated *in vivo* passaging [52]. *H. pylori* was administered to C3H/HeN mice and allowed to colonize the gut; subsequently pure colonies of *H. pylori* were isolated on EYE selective media. This step was repeated for 10 passages. During each passage, the MICs of TP4 and the conventional antibiotics were determined. All procedures, care, and handling of mice were approved by the laboratory animal ethics committee of National Taiwan Ocean University.

### Time-dependent efficacy of TP4 on multidrug resistant *H. pylori*

Overnight cultures of *H. pylori* were scraped and adjusted to OD 0.05, and 1 ml/tube inocula were prepared with BHI broth. Solutions of TP4 or clinical antibiotics were prepared with concentrations corresponding to their respective MIC, and PBS was used as a control. The tubes were incubated without shaking at 37°C under microaerophilic conditions, and sample aliquots were taken at 0, 1, 3, 6, 12, and 24 hr. The samples were serially diluted, and appropriate dilutions were plated in triplicate onto blood agar plates for bacterial counts. The results were expressed as mean log (c.f.u. mL^−1^) with standard error.

### Membrane perturbation assay based on N-phenyl-naphthylamine (NPN) uptake

The NPN membrane permeation assay was performed as described previously [66]. Briefly, a starter culture of *H. pylori* was grown overnight to 0.05 OD. The adjusted culture was aliquoted into different tubes with or without TP4/amoxicillin, and incubated at 37°C for 360 minutes under microaerophilic conditions. The cells were washed and resuspended in PBS to a final volume of 250 μl in the presence of 22μg mL^−1^ of NPN. Tubes containing PBS with NPN and bacterial cells with NPN served as controls. NPN fluorescence intensity was recorded using a Synergy HT (BioTek, U.S.A) fluorescence ELISA reader, and correlated to membrane potential and permeation. Experiments were performed in triplicate, and the results are expressed as means with standard error.

### Effect of TP4 on *H. pylori* surface charge

Zeta potential studies were performed at room temperature using a Zetasizer Nano ZS (Malvern Instruments, Worcestershire, UK) equipped with a 633-nm HeNe laser [36]. TP4 at the MIC was prepared. A 100 μl volume of each peptide stock dilution was added to 900 μl (0.5 OD) of *H. pylori* in liquid culture/blood agar cultures. Positive controls contained filtered buffer instead of peptide. The bacterial suspensions were dispensed into disposable zeta cells with gold electrodes, and allowed to equilibrate for 15 min at 25 °C. The zeta potential for each sample was calculated. The complete experiment was carried out twice for each peptide using independently-grown cultures.

### Transmission electron microscopy (TEM) analysis of TP4-induced morphological changes in *H. pylori*

Transmission electron microscopy (TEM) was used to examine the mechanism of TP4 action on *H. pylori*, overall cell morphology, TP4 translocation into *H. pylori*, and binding of TP4 to internal constituents. *H. pylori* overnight cultures from BHI-blood agar plates were collected, and 0.1 OD inocula were incubated with TP4, Amoxicillin, and/or PBS (PBS only: negative control). Following drug exposure, *H. pylori* cells were collected and successively washed twice in PBS by centrifugation and resuspension. The cells were then fixed and processed for TEM using previously described methods [67, 68]. The morphology of the cells was observed using an FEI Tecnai G2 F20 S-TWIN transmission electron microscope (FEI Company, Hillsboro, OR) operating at 80 keV [69] at low-power magnification (X1100), and the cell wall ultrastructure was observed at high-power magnification (X2, 500/5,000/7,000, or X15, 000). Each grid was examined under the same settings. Images were recorded with a 4MP SPOT Insight charge-coupled-device (CCD) camera.

### Monotherapeutic efficacy of TP4 against *H. pylori* infection in a mouse model

Male C3H/HeN mice were obtained from BioLASCO Tawian, co., Ltd., and housed at the Laboratory Animal Facility, National Taiwan Ocean University, Taiwan. Mice were maintained in pathogen-free sterile isolators, and all food, water, caging, and bedding were sterilized before use. All animal protocols with reference number 96025 were approved by the Institutional Animal Care and Use Committee (IACUC) of the College of Science, National Taiwan Ocean University. For the *H. pylori* infection model, six-week-old male mice were randomly divided into three groups of six mice each. To establish primary *H. pylori* infection, mice were intragastrically challenged with ~1×10^9^ c.f.u. of *H. pylori* on two days, with an interval of 24 hr [70]. After colonization (day 9 onwards), the mice were treated with a monotherapeutic dose of TP4 (8 mg/kg) every day for 14 days; the control group received an equivalent volume of PBS. Mice were sacrificed 2 wk post-treatment on day 22, and the gastric tissue was processed for urease activity, quantification of *H. pylori*, Western blot, histopathology analysis, T cell subset quantification, and gene expression analysis.

### Assessment of *H. pylori* colonization

Harvested stomach lysates were prepared and serially diluted, and 100 μl aliquots were spread onto EYE selective agar plates in duplicate. The plates were incubated under microaerophilic conditions at 37°C for 24-72 hr. Pink colonies (*H. pylori*) and total viable counts were recorded.

### Expression of *H. pylori* virulence factor genes

Genomic DNA (gDNA) was isolated from gastric tissue lysates (25 μl) using the Tissue and Cell Genomic DNA Purification Kit (GeneMark; DP021-50, Taiwan). The concentration and quality of the DNA was determined using a NanoDrop N1000 spectrophotometer. Polymerase chain reaction (PCR) was performed using gene specific primers (*UreB* F: 5′-GGC ACC ACT CCT TCT GCA AT-3′ R: 5′-CAG CTG TTT GCC AAG TTC TGG-3′, *CagA* F: 5′-GAT GTG AAA TCC CCG GGC TC-3′, R: 5′-ACT GCG ATC CGG ACT ACG AT-3′, internal control 16s rRNA F: 5′-ACG CGT CGA CAG AGT TTG ATC CTG GCT-3′ R: 5′-AGG CCC GGG AAC GTA TTC AC-3′ [71]) and 100 ng of gDNA as template. The resulting PCR products were separated by electrophoresis on a 2 % agarose gel, and then stained with ethidium bromide [44].

### Western blotting

Urease B protein was used as a marker to confirm the presence of *H. pylori* in gastric tissue. Total lysate protein (25 μg) was loaded onto a SDS gel and separated by electrophoresis. The protein bands were then transferred onto a PVDF membrane. After blotting, the membrane was blocked with 3% BSA in TBS with 0.01% Tween 20, and proteins were detected using Urease B specific antibody. A chemiluminescence detector (UVP BioSpectrum) was used to determine the relative intensity of the protein bands.

### Gastric tissue analyses

Gastric tissue biopsies were fixed in buffered paraffin and embedded in paraffin wax. A section of about 5 μm was stained with hematoxylin and eosin to analyze tissue inflammation. For staining against *H. pylori*, tissue slides were deparaffinized with xylene and alcohol, and then rehydrated in water. After washing in PBS, the tissue section was incubated with modified Giemsa stain for 2-5 minutes, and then washed with PBS. Slides were fixed, and sections were observed at different magnifications under light microscopy. The tissues were evaluated as described previously [72].

### Flow cytometry

Flow cytometry was performed to quantify mice splenic T cell subsets [73, 74]. Briefly, splenectomies were performed on euthanized mice, and the spleens were transferred into RPMI media. Spleens were minced and passed through a 100 μl size mesh at room temperature, and the single cell suspensions were pelleted at 1500 rpm for 5 min. The resulting cell pellet was resuspended in 3 ml of RBC lysis buffer, and incubated for 5 min at room temperature (RT) with gentle tapping to assist in lysis and cell disintegration; the reaction was halted by the addition of excess RPMI media. The mixture was centrifuged, and the pellet was resuspended in RPMI media. The resulting single cell suspension was aliquoted into flow cytometry tubes (100 μl/tube). One microliter of fluorescent dye-labeled monoclonal antibody (T-cell subsets; CD4, Th17, Treg) was added to each tube, and the volume was made up to 500 μl with PBS. The tubes were covered with foil and incubated in the dark for 1 hr at room temperature. A BD FACS Canto-A flow cytometer was used to record the percentages of T cell subsets.

### Gastric cytokines and T cell marker gene expression

Total RNA from stomachs of C3H/HeN mice was prepared using the High Pure RNA Tissue Kit according to the recommendations of the manufacturer (Roche, USA). For cytokine mRNA quantification, 5 μg of total RNA was converted into cDNA using a high capacity cDNA archive kit (Invitrogen). Levels of interleukin IL-1β, IL-6, IL-18, IL-10, IL-23, IL-17, tumor necrosis factor alpha (TNF –α), TGF-β, and FoxP3 mRNA were measured by Q-PCR using TaqMan gene expression assays for use in the A CFX Connect™ Real-Time PCR Detection System (Bio-Rad). Transcript levels were normalized to those of mRNA of the endogenous control glyceraldehyde-3-phosphate dehydrogenase (GAPDH), and expressed as the fold change compared to samples from control mice using the Comparative CT method (Bio-Rad).

### Toxicity studies of TP4

**Oral toxicity**: Acute oral toxicity was assessed in C3H/HeN mice with 6 animals per group, by single oral administration of TP4 (125 mg/kg) [47, 75]. After dose administration, the animals were kept under observation for a minimum of 48 hr. The mortality rate and clinical signs were recorded. Sub-acute oral toxicity was assessed by administering 600 μg dose/day for 14 days [48]. During dose administration, the animals were observed every day for any signs of toxicity. After the treatment period, the animals were kept under observation for 14 days. The morbidity, mortality, and other clinical signs were recorded and assessed. Dermal Toxicity was assessed by topical application of TP4 at 10 times the effective dose to skin at the dorsal side of mice [50, 76]. After administration, the animals were kept under observation for a minimum of 48 h. Clinical signs were noted if the animals were in the morbid stage or presented with any abnormalities. Eye irritation test in New Zealand white rabbits: Healthy young adult rabbits were used in the experiment. Eyes were treated with TP4, PBS (vehicle control), or 0.1% SDS (positive control). A repeated dose of 1 mg/kg was administered every day for 7 days. During the treatment period, the eyes were examined on days 1, 3, 5, and 7, and allowed to recover until day 14. Clinical signs were recorded during the test period, and grades were assigned for ocular reactions observed during each examination [49]. All procedures, care, and handling of rabbits were approved by the laboratory animal ethics committee of National Taiwan Ocean University.

### Statistical analysis

Statistical analyses were performed and graphs were generated using SPSS 17.0, Graphpad 5.2 software. Results are represented as means ± s.e.m. (standard error of the mean). Statistical analyses were performed using Student's t–test, ANOVA. The criterion for significance was set to p < 0.05.

## Abbreviations

TP4: Tilapia piscidin 4
AOMX: amoxicillin
CLRN: clarithromycin
METZ: metronidazole
PPI: proton pump inhibitor
ST: sequential therapy
RUT: rapid urea test

## References

[1] Marshall BJ, Warren JR (1984). Unidentified curved bacilli in the stomach of patients with gastritis and peptic ulceration. Lancet.

[2] Nomura A, Stemmermann GN, Chyou PH, Perez-Perez GI, Blaser MJ (1994). *Helicobacter pylori* infection and the risk for duodenal and gastric ulceration. Ann Intern Med.

[3] Nomura A, Stemmermann GN, Chyou P-H, Kato I, Perez-Perez GI, Blaser MJ (1991). *Helicobacter pylori* infection and gastric carcinoma among Japanese Americans in Hawaii. N Engl J Med.

[4] Parsonnet J, Hansen S, Rodriguez L, Gelb AB, Warnke RA, Jellum E, Orentreich N, Vogelman JH, Friedman GD (1994). *Helicobacter pylori* infection and gastric lymphoma. N Engl J Med.

[5] Kusters JG, van Vliet AH, Kuipers EJ (2006). Pathogenesis of *Helicobacter pylori* infection. Clin Microbiol Rev.

[6] Lee YC, Liou JM, Wu MS, Wu CY, Lin JT (2008). Eradication of *Helicobacter pylori* to prevent gastroduodenal diseases: hitting more than one bird with the same stone. Therap Adv Gastroenterol.

[7] Correa P, Houghton J (2007). Carcinogenesis of *Helicobacter pylori*. Gastroenterology.

[8] Hashim H, Azmin S, Razlan H, Yahya NW, Tan HJ, Manaf MR, Ibrahim NM (2014). Eradication of *Helicobacter pylori* infection improves levodopa action, clinical symptoms and quality of life in patients with Parkinson's disease. PLoS One.

[9] Dobbs RJ, Dobbs SM, Weller C, Bjarnason IT, Oxlade NL, Charlett A, Al-Janabi MA, Kerwin RW, Mahler RF, Price AB (2005). Role of chronic infection and inflammation in the gastrointestinal tract in the etiology and pathogenesis of idiopathic parkinsonism. Helicobacter.

[10] Altschuler E (1996). Gastric *Helicobacter pylori* infection as a cause of idiopathic parkinson disease and non-arteric anterior optic ischemic neuropathy. Med Hypotheses.

[11] Khoshnood A, Hakimi P, Salman-Roghani H, Reza Mirjalili M (2014). Replacement of clarithromycin with azithromycin in triple therapy regimens for the eradication of *Helicobacter pylori*: A randomized clinical trial. J Med Life.

[12] Marin AC, McNicholl AG, Gisbert JP (2013). A review of rescue regimens after clarithromycin-containing triple therapy failure (for *Helicobacter pylori* eradication). Expert Opin Pharmacother.

[13] Mirzaei N, Poursina F, Moghim S, Rahimi E, Safaei HG (2014). The mutation of the rdxA gene in metronidazole-resistant *Helicobacter pylori* clinical isolates. Adv Biomed Res.

[14] Malfertheiner P, Megraud F, O'Morain C, Bazzoli F, El-Omar E, Graham D, Hunt R, Rokkas T, Vakil N, Kuipers EJ (2007). Current concepts in the management of *Helicobacter pylori* infection: the Maastricht III Consensus Report. Gut.

[15] Brogden KA (2005). Antimicrobial peptides: pore formers or metabolic inhibitors in bacteria?. Nat Rev Microbiol.

[16] Ellison RT, Giehl TJ, LaForce FM (1988). Damage of the outer membrane of enteric gram-negative bacteria by lactoferrin and transferrin. Infect Immun.

[17] Viejo-Diaz M, Andres MT, Fierro JF (2005). Different anti-Candida activities of two human lactoferrin-derived peptides, Lfpep and kaliocin-1. Antimicrob Agents Chemother.

[18] Radzishevsky I, Krugliak M, Ginsburg H, Mor A (2007). Antiplasmodial activity of lauryl-lysine oligomers. Antimicrob Agents Chemother.

[19] Herce HD, Garcia AE, Litt J, Kane RS, Martin P, Enrique N, Rebolledo A, Milesi V (2009). Arginine-rich peptides destabilize the plasma membrane, consistent with a pore formation translocation mechanism of cell-penetrating peptides. Biophys J.

[20] Huang HN, Pan CY, Chan YL, Chen JY, Wu CJ (2014). Use of the Antimicrobial peptide pardaxin (GE33) to protect against methicillin-resistant *Staphylococcus aureus* infection in mice with skin injuries. Antimicrob Agents Chemother.

[21] Peters BM, Shirtliff ME, Jabra-Rizk MA (2010). Antimicrobial peptides: primeval molecules or future drugs?. PLoS Pathog.

[22] Guani-Guerra E, Santos-Mendoza T, Lugo-Reyes SO, Teran LM (2010). Antimicrobial peptides: general overview and clinical implications in human health and disease. Clin Immunol.

[23] Zasloff M (1987). Magainins, a class of antimicrobial peptides from Xenopus skin: isolation, characterization of two active forms, and partial cDNA sequence of a precursor. Proc Natl Acad Sci U S A.

[24] Sader HS, Fedler KA, Rennie RP, Stevens S, Jones RN (2004). Omiganan pentahydrochloride (MBI 226), a topical 12-amino-acid cationic peptide: spectrum of antimicrobial activity and measurements of bactericidal activity. Antimicrob Agents Chemother.

[25] Silphaduang U, Noga EJ (2001). Peptide antibiotics in mast cells of fish. Nature.

[26] Noga EJ, Silphaduang U, Park NG, Seo JK, Stephenson J, Kozlowicz S (2009). Piscidin 4, a novel member of the piscidin family of antimicrobial peptides. Comp Biochem Physiol B Biochem Mol Biol.

[27] Lee SA, Kim YK, Lim SS, Zhu WL, Ko H, Shin SY, Hahm KS, Kim Y (2007). Solution structure and cell selectivity of piscidin 1 and its analogues. Biochemistry.

[28] Sung WS, Lee J, Lee DG (2008). Fungicidal effect and the mode of action of piscidin 2 derived from hybrid striped bass. Biochem Biophys Res Commun.

[29] Peng KC, Lee SH, Hour AL, Pan CY, Lee LH, Chen JY (2012). Five different piscidins from Nile tilapia, *Oreochromis niloticus*: analysis of their expressions and biological functions. PLoS One.

[30] Winkler ML, Papp-Wallace KM, Hujer AM, Domitrovic TN, Hujer KM, Hurless KN, Tuohy M, Hall G, Bonomo RA (2015). Unexpected challenges in treating multidrug-resistant gram-negative rods: resistance to ceftazidime-avibactam in archived isolates of *Pseudomonas aeruginosa*. Antimicrob Agents Chemother.

[31] Khara JS, Wang Y, Ke XY, Liu S, Newton SM, Langford PR, Yang YY, Ee PL (2014). Anti-mycobacterial activities of synthetic cationic alpha-helical peptides and their synergism with rifampicin. Biomaterials.

[32] Urban C, Mariano N, Rahal JJ (2010). *In vitro* double and triple bactericidal activities of doripenem, polymyxin B, and rifampin against multidrug-resistant *Acinetobacter baumannii, Pseudomonas aeruginosa, Klebsiella pneumoniae*, and *Escherichia coli*. Antimicrob Agents Chemother.

[33] Nishizawa T, Suzuki H, Suzuki M, Takahashi M, Hibi T (2012). Proton pump inhibitor-amoxicillin-clarithromycin versus proton pump inhibitor-amoxicillin-metronidazole as first-line *Helicobacter pylori* eradication therapy. J Clin Biochem Nutr.

[34] Sarig H, Goldfeder Y, Rotem S, Mor A (2011). Mechanisms mediating bactericidal properties and conditions that enhance the potency of a broad-spectrum oligo-acyl-lysyl. Antimicrob Agents Chemother.

[35] Makobongo MO, Gancz H, Carpenter BM, McDaniel DP, Merrell DS (2012). The oligo-acyl lysyl antimicrobial peptide C12K-2β12 exhibits a dual mechanism of action and demonstrates strong *in vivo* efficacy against *Helicobacter pylori*. Antimicrob Agents Chemother.

[36] Alves CS, Melo MN, Franquelim HG, Ferre R, Planas M, Feliu L, Bardaji E, Kowalczyk W, Andreu D, Santos NC, Fernandes MX, Castanho MA (2010). *Escherichia coli* cell surface perturbation and disruption induced by antimicrobial peptides BP100 and pepR. J Biol Chem.

[37] Berry V, Jennings K, Woodnutt G (1995). Bactericidal and morphological effects of amoxicillin on *Helicobacter pylori*. Antimicrob Agents Chemother.

[38] Bode G, Mauch F, Malfertheiner P (1993). The coccoid forms of *Helicobacter pylori*. criteria for their viability. Epidemiol. Infect.

[39] Müller M, dela Peña A, Derendorf H (2004). Issues in pharmacokinetics and pharmacodynamics of anti-infective agents: distribution in tissue. Antimicrob Agents Chemother.

[40] Aoki W, Ueda M (2013). Characterization of antimicrobial peptides toward the development of novel antibiotics. Pharmaceuticals (Basel).

[41] Li H, Cheng JW, Yu HY, Xin Y, Tang L, Ma Y (2013). Effect of the antimicrobial peptide D-Nal-Pac-525 on the growth of *Streptococcus mutans* and its biofilm formation. J Microbiol. Biotechn..

[42] Xiang Z, Censini S, Bayeli PF, Telford JL, Figura N, Rappuoli R, Covacci A (1995). Analysis of expression of CagA and VacA virulence factors in 43 strains of *Helicobacter pylori* reveals that clinical isolates can be divided into two major types and that CagA is not necessary for expression of the vacuolating cytotoxin. Infect Immun.

[43] Shahamat M, Alavi M, Watts JE, Gonzalez JM, Sowers KR, Maeder DW, Robb FT (2004). Development of two PCR-based techniques for detecting helical and coccoid forms of *Helicobacter pylori*. J Clin Microbiol.

[44] Rimbara E, Sasatsu M, Graham DY (2013). PCR detection of *Helicobacter pylori* in clinical samples. Methods Mol Biol (Clifton, NJ).

[45] Rad R, Brenner L, Bauer S, Schwendy S, Layland L, da Costa CP, Reindl W, Dossumbekova A, Friedrich M, Saur D, Wagner H, Schmid RM, Prinz C (2006). CD25+/Foxp3+ T cells regulate gastric inflammation and *Helicobacter pylori* colonization *in vivo*. Gastroenterology.

[46] Kao JY, Zhang M, Miller MJ, Mills JC, Wang B, Liu M, Eaton KA, Zou W, Berndt BE, Cole TS, Takeuchi T, Owyang SY, Luther J (2010). *Helicobacter pylori* immune escape is mediated by dendritic cell-induced Treg skewing and Th17 suppression in mice. Gastroenterology.

[47] OECD Test No. 423: Acute oral toxicity - acute toxic class method.

[48] OECD Test No. 407: Repeated dose 28-day oral toxicity study in rodents.

[49] OECD Test No. 405: Acute eye irritation/corrosion.

[50] OECD Test No. 406: Skin sensitisation.

[51] Jones KR, Cha JH, Merrell DS (2008). Who's winning the war? molecular mechanisms of antibiotic resistance in *Helicobacter pylori*. Curr Drug Ther.

[52] Iwao E, Yamamoto K, Yokoyama Y, Hirayama F, Haga K (2004). Potent antibacterial activity of Y-754, a novel benzimidazole compound with selective action against *Helicobacter pylori*. J Infect Chemother.

[53] McGee DJ, George AE, Trainor EA, Horton KE, Hildebrandt E, Testerman TL (2011). Cholesterol enhances *Helicobacter pylori* resistance to antibiotics and LL-37. Antimicrob Agents Chemother.

[54] Van Amsterdam K, Bart A, van der Ende A (2005). A *Helicobacter pylori* tolc efflux pump confers resistance to metronidazole. Antimicrob Agents Chemother.

[55] Hurdle JG, O'Neill AJ, Chopra I, Lee RE (2011). Targeting bacterial membrane function: an underexploited mechanism for treating persistent infections. Nat Rev Microbiol.

[56] Pan CY, Chen JC, Sheen JF, Lin TL, Chen JY (2014). Epinecidin-1 has immunomodulatory effects, facilitating its therapeutic use in a mouse model of *Pseudomonas aeruginosa* sepsis. Antimicrob Agents Chemother.

[57] Fox JL (2013). Antimicrobial peptides stage a comeback. Nat Biotechnol.

[58] Chan DI, Prenner EJ, Vogel HJ (2006). Tryptophan- and arginine-rich antimicrobial peptides: structures and mechanisms of action. Biochimica et biophysica acta.

[59] Rollema HS, Kuipers OP, Both P, de Vos WM, Siezen RJ (1995). Improvement of solubility and stability of the antimicrobial peptide nisin by protein engineering. Appl Environ Microbiol.

[60] Tu Z, Young A, Murphy C, Liang JF (2009). The pH sensitivity of histidine-containing lytic peptides. J. Pept. Sci.

[61] Gray BM, Fontaine CA, Poe SA, Eaton KA (2013). Complex T cell interactions contribute to *Helicobacter pylori* gastritis in mice. Infect Immun.

[62] Shi Y, Liu XF, Zhuang Y, Zhang JY, Liu T, Yin Z, Wu C, Mao XH, Jia KR, Wang FJ, Guo H, Flavell RA, Zhao Z, Liu KY, Xiao B, Guo Y (2010). *Helicobacter pylori* -induced Th17 responses modulate Th1 cell responses, benefit bacterial growth, and contribute to pathology in mice. J Immunol.

[63] Makobongo MO, Kovachi T, Gancz H, Mor A, Merrell DS (2009). In vitro antibacterial activity of acyl-lysyl oligomers against *Helicobacter pylori*. Antimicrob Agents Chemother.

[64] Lin WJ, Chien YL, Pan CY, Lin TL, Chen JY, Chiu SJ, Hui CF (2009). Epinecidin-1, an antimicrobial peptide from fish (*Epinephelus coioides*) which has an antitumor effect like lytic peptides in human fibrosarcoma cells. Peptides.

[65] Huang H-N, Rajanbabu V, Pan C-Y, Chan Y-L, Wu C-J, Chen J-Y (2013). Use of the antimicrobial peptide Epinecidin-1 to protect against MRSA infection in mice with skin injuries. Biomaterials.

[66] Lv Y, Wang J, Gao H, Wang Z, Dong N, Ma Q, Shan A (2014). Antimicrobial properties and membrane-active mechanism of a potential alpha-helical antimicrobial derived from cathelicidin PMAP-36. PLoS One.

[67] Xie M, Tobin JE, Budde MD, Chen CI, Trinkaus K, Cross AH, McDaniel DP, Song SK, Armstrong RC (2010). Rostrocaudal analysis of corpus callosum demyelination and axon damage across disease stages refines diffusion tensor imaging correlations with pathological features. J Neuropath Exp Neur.

[68] McDaniel DP, Roberson RW (2000). Microtubules are required for motility and positioning of vesicles and mitochondria in hyphal tip cells of *Allomyces macrogynus*. Fungal Genet Biol.

[69] Wu SP, Huang TC, Lin CC, Hui CF, Lin CH, Chen JY (2012). Pardaxin, a fish antimicrobial peptide, exhibits antitumor activity toward murine fibrosarcoma *in vitro* and *in vivo*. Mar Drugs.

[70] Obonyo M, Guiney DG, Harwood J, Fierer J, Cole SP (2002). Role of gamma interferon in *Helicobacter pylori* induction of inflammatory mediators during murine infection. Infect Immun.

[71] James G (2010). Universal bacterial identification by PCR and DNA sequencing of 16S rRNA Gene.

[72] Sobala GM, Crabtree JE, Dixon MF, Schorah CJ, Taylor JD, Rathbone BJ, Heatley RV, Axon AT (1991). Acute *Helicobacter pylori* infection: clinical features, local and systemic immune response, gastric mucosal histology, and gastric juice ascorbic acid concentrations. Gut.

[73] Colovai AI, Giatzikis C, Ho EK, Farooqi M, Suciu-Foca N, Cattoretti G, Orazi A (2004). Flow cytometric analysis of normal and reactive spleen. Mod Pathol.

[74] Wang Z, Friedrich C, Hagemann SC, Korte WH, Goharani N, Cording S, Eberl G, Sparwasser T, Lochner M (2014). Regulatory T cells promote a protective Th17-associated immune response to intestinal bacterial infection with *C. rodentium*. Mucosal Immunol.

[75] de Almeida Vaucher R, de Campos Velho Gewehr C, Folmer Correa AP, Sant‘Anna V, Ferreira J, Brandelli A (2011). Evaluation of the immunogenicity and toxicity of the antimicrobial peptide P34. Int. J. Pharm.

[76] Sekizawa J, Yasuhara K, Suyama Y, Yamanaka S, Tobe M, Nishimura M (1994). A simple method for screening assessment of skin and eye irritation. J Toxicol Sci.

